# Comprehensive Assessment of Inactivation Methods for Madariaga Virus

**DOI:** 10.3390/v16020206

**Published:** 2024-01-30

**Authors:** RuthMabel Boytz, Kadiatou Keita, Joanna B Pawlak, Maudry Laurent-Rolle

**Affiliations:** 1 Department of Microbial Pathogenesis, Yale University School of Medicine, New Haven, CT 06520, USA; ruthmabel.boytz@yale.edu; 2 Section of Infectious Diseases, Department of Internal Medicine, Yale University School of Medicine, New Haven, CT 06520, USA; k.keita@yale.edu (K.K.); joanna.pawlak@yale.edu (J.B.P.); 3 Center for Infection and Immunity, Yale University School of Medicine, New Haven, CT 06520, USA

**Keywords:** Madariaga, Eastern Equine Encephalitis Virus, inactivation, protocol, Select Agent

## Abstract

The Eastern Equine Encephalitis Virus (EEEV) is an emerging public health threat, with the number of reported cases in the US increasing in recent years. EEEV is a BSL3 pathogen, and the North American strain is a US Federal Select Agent (SA). These restrictions make experiments with EEEV difficult to perform, as high-tech equipment is often unavailable in BSL3 spaces and due to concerns about generating aerosols during manipulations. Therefore, a range of inactivation methods suitable for different downstream analysis methods are essential for advancing research on EEEV. We used heat, chemical, and ultraviolet (UV)-based methods for the inactivation of infected cells and supernatants infected with the non-select agent Madariaga virus (MADV). Although the MADV and EEEV strains are genetically distinct, differing by 8–11% at the amino acid level, they are expected to be similarly susceptible to various inactivation methods. We determined the following to be effective methods of inactivation: heat, TRIzol LS, 4% PFA, 10% formalin, and UV radiation for infected supernatants; TRIzol, 2.5% SDS with BME, 0.2% NP40, 4% PFA, and 10% formalin for infected cells. Our results have the potential to expand the types and complexity of experiments and analyses performed by EEEV researchers.

## 1. Background

Eastern Equine Encephalitis Virus (EEEV) is an alphavirus in the *Togaviridae* family and is primarily maintained in an enzootic cycle between *Culiseta melanura* (Coquillett) mosquitoes and birds [1,2,3]. There are four antigenic subtypes of EEEV. One subtype comprises strains from North America, and the remaining three antigenic subtypes are found in Central and South America [3,4]. These subtypes exhibit important differences in their virulence. In general, North American EEEV strains are an uncommon cause of meningoencephalitis in humans, with mortality as high as 35% in symptomatic patients and severe neurological sequelae in those who survive [5,6,7]. There are no approved US Food and Drug Administration antivirals, and there are no approved vaccines for human use. EEEV can be transmitted as an aerosol, therefore it can be weaponized for bioterrorism [8,9,10], and it is regulated under the U.S. Federal Select Agent Program. There were 38 cases of North American strain EEEV (henceforth EEEV) reported in the US in 2019, compared to an average of 6.4 cases per year in the five preceding years [11]. The most affected states include Florida, New Hampshire, Michigan, North Carolina, and Georgia [11]. Changes in temperature and precipitation patterns associated with climate change are expected to increase the range of vector species, placing more of the population at risk for EEEV [12,13]. Therefore, EEEV represents an emerging threat to public health, and basic research on EEEV is essential.

Due to space restrictions, challenges of maintaining high-tech equipment in a BSL3 lab containing select agents, and concerns about generating aerosols during experiential manipulation, high-tech equipment is limited within the BSL3 space. To perform downstream analyses on experimental samples and to protect researchers from infection, a variety of different inactivation methods are required. There are few published inactivation methods specifically for EEEV, and any procedures must be validated in-house in accordance with CDC select agent guidelines. Madariaga virus (MADV) is the South American strain of EEEV. It shares 89–92% amino acid identity (approximately 76% nucleotide identity) with the North American EEEV [4] and is expected to have the same susceptibility to inactivation methods. We used MADV as a surrogate virus for EEEV. We have tested the efficacy of 10% formalin, 4% PFA, TRIzol, TRIzol LS, 0.2% NP40, 2.5% SDS, heat, and UV for inactivating supernatants and cells infected with MADV.

## 2. Methods

### 2.1. Virus Propagation

A stock of Madariaga virus, BeAr348998, originally isolated in Brazil and obtained from the Connecticut Agricultural Experiment Station (New Haven, CT, USA), was initially grown on BHK-21 cells to generate a working stock. A plaque-purified isolate was propagated on BHK-21 cells, and the identity of the virus as MADV was confirmed by sequencing. This stock, denoted MADV 1, had a titer of 6.6 × 10^7^ plaque-forming units per mL (PFU/mL) and was used in all inactivation studies. All work with live viruses was approved on 27 March 2023 by Yale’s Environmental Health and Safety Department and performed in the Yale Biosafety Level 3 laboratory suite (Protocol number 350809).

### 2.2. Plaque Assays

**Figure 1:**
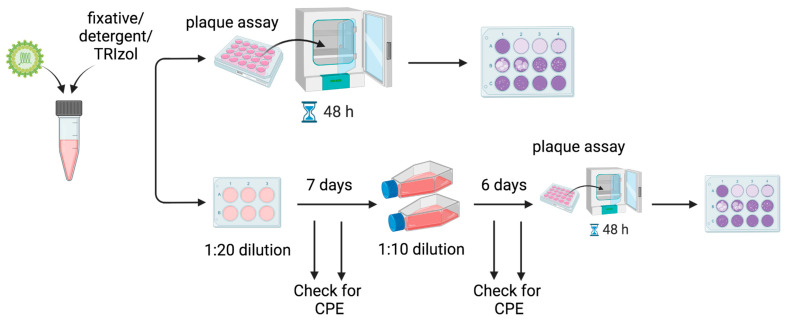
Overview of workflow. MADV was inactivated by various methods, and viable virus was detected using plaque assays on BHK-21 cells. Following inactivation, samples were tested for successful inactivation by a 48 h plaque assay, which had a limit of detection of 6.6 × 10^−2^ PFU/mL, or 50 virions per mL (Top). Due to cell loss from detergent or fixative alone, and to increase the sensitivity of detection of viable viruses, a 13-day double passage experiment was also performed (Bottom). After inactivation, the sample was initially diluted 1:20 and placed onto fresh BHK-21 cells in 6-well plates. The cells were incubated for 7 days and monitored for the development of cytopathic effects (CPE), which would indicate the presence of live virus. After 7 days, a sample from each well was diluted 1:10 onto fresh BHK-21 cells in T75 flasks. The cells were incubated for another 6 days and monitored for CPE. After 6 days, plaque assays were performed to detect any remaining viable virus.

Plaque assays on BHK-21 cells were used to quantify viral titers and verify inactivation. BHK-21 cells were plated in 12-well plates overnight until they reached 80% confluency. The following day, the media was removed and replaced with 150 μL of diluted virus or test inactivation from a 10-fold serial dilution (10^−1^ to 10^−11^). Plates were incubated for 1 h at 37 °C with periodic rocking. After 1 h, the inoculum was removed and 1 mL of overlay was added (0.75% low-melting-point agarose (Invitrogen, Waltham, MA, USA), 45% MEM (Gibco Thermo Fisher, Waltham, MA, USA), and 5% FBS). The plates were kept at room temperature for 15 min until the overlay solidified, then moved to a 37 °C incubator with 5% CO_2_ for two days. After two days, the cells were fixed for 1 h in 1 mL of 10% formalin added directly to the overlay, and plaques were developed by adding crystal violet (1% crystal violet, 30% ethanol) and rinsing with water. A schematic is shown in Figure 1.

### 2.3. 13-Day, Two-Passage Experiments

Due to cell loss caused by the fixatives and detergents at 10^−1^ and 10^−2^ dilutions, plaque assays could not be used to detect viable viruses remaining at these dilutions following treatment. To detect the presence of the remaining viable virus, 100 μL of the test inactivation was added to BHK-21 cells in 6-well plates. The cells were incubated for 7 days at 37 °C in DMEM supplemented with 2% FBS. The wells were periodically monitored for cytopathic effects (CPE) using a microscope, which would indicate the presence of an infectious virus. After 7 days, 1 mL of the supernatant was transferred to fresh T75 flasks of BHK-21 cells in 10 mL DMEM (Gibco Thermo Fisher, Waltham, MA, USA) supplemented with 2% U.S. origin FBS. The flasks were incubated for an additional 6 days and monitored for CPE. After 6 days, plaque assays were performed to detect viable viruses. See Figure 1. The 13-day experiments were performed in biological duplicate.

### 2.4. Inactivations

All inactivations were performed in biological triplicates. Each plaque assay included an infected well of cells and a well of cells infected with an untreated virus diluted to 10^−5^. For each fixative, a treatment-only (i.e., uninfected media or cells plus the fixative) plaque assay was performed in parallel to control for cell loss due to the fixative alone.

### 2.5. Inactivation of Supernatants with TRIzol LS

The inactivation was performed in biological triplicates using 6.6 × 10^5^ PFU of MADV 1. A total of 100 μL of virus was mixed with 400 μL of TRIzol LS (Sigma Aldrich, St. Louis, MO, USA) in a screw-cap tube and incubated for 15 min at room temperature. Viable virus was detected using plaque assays and also assessed using the 13-day passage experiment.

### 2.6. Inactivation of Supernatants with Formalin and PFA

The inactivation was performed in biological triplicates using 6.6 × 10^5^ PFU of MADV 1. Fixatives were prepared in the PBS as follows: 13.3% formalin, 5.3% paraformaldehyde (PFA) (Sigma Aldrich, St. Louis, MO, USA). In a screw-cap tube, 100 μL of virus was mixed with 300 μL of fixative for a final concentration of 10% formalin or 4% PFA and incubated for 30 or 60 min at room temperature. Viable virus was detected using plaque assays and also assessed using the 13-day passage experiment.

### 2.7. Inactivation of Cells with TRIzol

One day prior to inactivation, BHK-21 cells in 6-well plates were infected with MADV-1 at an MOI of 1.0. The following day, the media was removed, and cells were washed with PBS. We added 1 mL of TRIzol (Sigma Aldrich, St. Louis, MO, USA) per well, or about 1 × 10^6^ cells. Cells were collected in screw-cap tubes and incubated at room temperature for 15 min. Inactivations were performed in biological triplicates. Viable virus was detected using plaque assays and also assessed using the 13-day passage experiment.

### 2.8. Inactivation of Cell Pellets with Formalin and PFA

Inactivations were performed in biological triplicates. One day prior to inactivation, BHK-21 cells in 6-well plates were infected with MADV-1 at an MOI of 1.0. The following day, the media was removed, and cells were washed with PBS. Cells were removed with a 0.25% trypsin EDTA solution (Gibco Thermo Fisher, Waltham, MA, USA), collected in a screw-cap tube, and pelleted at 300× *g* for 5 min. We resuspended the cells in 500 μL of 10% formalin or 4% PFA in PBS per well, about 1 × 10^6^ cells. The resuspended pellets were incubated for 60 min at room temperature, and viable virus was detected using plaque assays and also assessed using the 13-day passage experiment.

### 2.9. Inactivation of Cell Pellets with 2.5% SDS and 0.2% NP40

Inactivations were performed in biological triplicates. One day prior to inactivation, BHK-21 cells in 6-well plates were infected with MADV-1 at an MOI of 1.0. The following day, the media was removed, and cells were trypsinized, pelleted, and washed with PBS. We added 1 mL of a lysis buffer containing 0.2% NP40 (Sigma Aldrich, St. Louis, MO, USA) or 500 μL of 2× Laemmli lysis buffer (BioRad, Hercules, CA, USA) containing 2.5% sodium dodecyl sulfate (SDS) with 2-mercaptoethanol (BME) to resuspend the cell pellets in screw-cap tubes. These were incubated on ice for 30 min, and viable virus was detected using plaque assays and also assessed using the 13-day passage experiment.

### 2.10. Inactivation of the Supernatant with an Ultraviolet Radiation Linker

A total of 500 μL of viral stock was added to each well of a 6-well plate. The plates were placed in a BioRad UV Genelinker (BioRad, Hercules, CA, USA). The plates were placed on the bottom interior surface of the Genelinker, and the plate lid was removed. The following settings were assessed: two cycles of 999 mJoule, one cycle of 999 mJoule, one cycle of 250 mJoule, and one cycle of 90 s. Inactivations were performed in biological triplicates, and plaque assays were used to assess viral inactivation.

### 2.11. Inactivation of the Supernatant with Heat

A total of 200 μL of viral stock was incubated at 95 °C in a heat block for 5, 10, or 15 min. Inactivations were performed in biological triplicates, and plaque assays were used to assess viral inactivation.

### 2.12. Western Blot Analysis

Ultraviolet (UV)- and formalin-inactivated supernatants were analyzed for E2 protein expression by SDS-PAGE and Western blotting. For lysis, an equal volume of inactivated supernatant and lysis buffer (0.5% NP40, 50 mM) Tris pH 8.0, 280 mM NaCl, 0.2 mM EDTA, 10% glycerol, 1 mM sodium orthovanadate, and an EDTA-free protease inhibitor cocktail (Roche, Basel, Switzerland) were incubated on ice for 20 min. Lysed and un-lysed samples were then diluted 1:1 in 2× Laemmli loading buffer (BioRad, Hercules, CA, USA) containing BME, boiled for 10 min, and separated on Mini-PROTEAN TGX precast gels (BioRad, Hercules, CA, USA) with tris-glycine running buffer containing 0.1% SDS. Proteins were transferred to PVDF membranes using the Trans-Blot Turbo transfer system (Bio Rad, Hercules, CA, USA). The membrane was blocked for 1 h at room temperature in TBS + 0.1% Tween-20 (TBS-T) containing 5% BSA with gentle rocking and incubated with rabbit anti-EEEV E2 glycoprotein primary antibody (IBT Bioservices, Rockville, MD, USA) diluted 1:1000 in 5% BSA TBS-T overnight at 4 °C with gentle rocking. The blot was washed three times in TBS-T, followed by incubation for 2 h at room temperature with goat-anti-rabbit HRP-conjugated secondary antibody (Jackson Immunoresearch 111-035-144) diluted 1:5000 in 1% BSA TBS-T. Blots were washed three times, developed with SuperSignal West Pico PLUS chemiluminescent substrate (Thermo Scientific, Waltham, MA, USA), and imaged on a Chemidoc MP (Bio Rad, Hercules, CA, USA).

### 2.13. Reverse-Transcription PCR and Virus Sequencing

MADV was inactivated by TRIzol LS as described above, and RNA extraction was performed using the Zymo Research RNA Extraction Kit (Zymo Research, Irvine, CA, USA) following the manufacturer’s instructions. Complementary DNA (cDNA) was synthesized using the SuperScript IV Reverse Transcriptase Kit, using random hexamers and according to the manufacturer’s instructions (Thermofisher, Waltham, MA, USA). To amplify viral genomic material, an approximately 4kb DNA fragment (4022 bp) was amplified using Superfi II Polymerase with the following primers: MAD-F1-Forward ATAGGGTATGGTGTAGAGGCAGCCAC, MAD-F1-Reverse CCGGCCTCATACTGTGTGGAACC. The reaction mixture was annealed at 72 °C for optimal results. The virus was sequenced using the following primer: MAD-2664V CATAGATACCACGAGCACCACG. The identity of the virus was confirmed as Madariaga by running a BLAST search of the sequencing results.

## 3. Results

**Figure 2:**
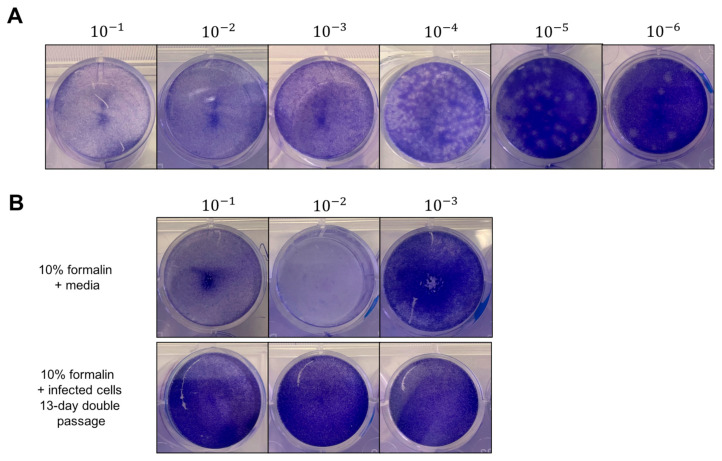
Viable virus was detected by plaque assay, but cell loss was observed due to formalin, detergent, or TRIzol alone. (**A**) Representative result of titration of initial stock MADV virus on BHK-21 cells. MADV was serially-diluted 1:10 fold and incubated for 1 h at 37 °C on BHK-21 cells with periodic rocking. Inoculum was removed and replaced with solid overlay media, and cells were incubated for a further 48 h at 37 °C. After fixation with 10% formalin for 1 h, plaques were developed with crystal violet, counted, and used to calculate viral titers. Shown is a representative plate, and the black lines indicate where separate images were taken of the wells. (**B**) Representative image showing cell loss from plaque assay plates due to formalin. Top panel shows cell loss due to formalin with media only at dilutions 10^−1^ and 10^−2^. Bottom panel shows results after 13 days of double passage of formalin-treated infected cells.

We tested heat inactivation, formaldehyde fixatives, detergents, TRIzol, and UV-based methods for the inactivation of MADV-infected supernatants and cells. For formaldehyde fixative-based methods, we treated samples of stock virus (in DMEM supplemented with 2% FBS) for 30 or 60 min with 10% formalin or 4% PFA, or infected BHK21 cell pellets for 60 min with 10% formalin or 4% PFA at room temperature. The supernatants contained 6.6 × 10^5^ total PFU of stock MADV 1, and the cell pellets contained 1 million cells and 6.6 × 10^10^ total PFU of MADV 1. After inactivation, we used plaque assays on BHK-21 cells to detect the remaining viable virus. We made a serial dilution of each sample, beginning at 10^−1^ and ending at 10^−10^, and incubated the inoculum directly on BHK-21 cells for 1 h, after which the inoculum was removed, replaced with a solid overlay, and incubated for a further 48 h. No plaques were observed after treatment with 10% formalin or 4% PFA for 30 min or 60 min (Table 1). However, uninfected controls (i.e., media only) mixed with formalin or PFA resulted in cell loss from the plaque assays at the first two dilutions in the series (i.e., 10^−1^ and 10^−2^) (Figure 2B). By the third serial dilution (10^−3^), the fixative was dilute enough to not cause cell death during the initial 1 h incubation. This pattern of cell loss at the two dilutions where the fixative concentration was greatest was also observed with the infected samples. Therefore, if the inactivated samples contained viable viruses, we would be unable to detect them at these low dilutions. Without cell loss, our plaque assay procedure for detecting viable viruses has a limit of detection of 6.6 × 10^−2^ PFU/mL, or 50 virions per mL.

To improve the sensitivity of our assay and to rule out the possibility of remaining viable viruses that were missed due to fixative-induced cell loss, we performed a 13-day double-passage experiment (Figure 1). We added 100 μL of each experimental inactivation to fresh BHK21 cells in 6-well plates and incubated these samples for 7 days. After 7 days, we passaged 1 mL of the supernatant onto fresh BHK21 cells in T75 culture flasks. Finally, after 6 days, we performed our standard plaque assay on these supernatants. During the 13-day period, we monitored the plates and flasks for signs of virus-induced CPE. While we observed cells detached from the T75 flasks by day 6, this did not resemble virus-induced CPE and was likely due to the cells running out of nutrients in the growth media. By comparison, cells infected with MADV at MOI = 0.1 or 0.01 began detaching and showed clear indications of CPE by 48 h post-infection. Complete inactivation was confirmed by our plaque assays, which had zero plaques even at the initial dilution (Table 2). Importantly, after the 13-day double passage experiment, there was no cell loss at the 10^−1^ and 10^−2^ dilutions where cell loss had previously been observed in the initial plaque assays. Any fixative in the initial sample had been highly diluted by this time. Taken together, the initial plaque assay and the 13-day double passage experiment confirm that 10% formalin and 4% PFA fully inactivated both MADV-infected culture supernatants and cell pellets.

Detergent-based methods of inactivation are useful for collecting cell lysate samples for downstream protein analysis, for example, Western blotting. We therefore assessed whether 0.2% NP40 and 2.5% SDS could inactivate MADV-infected cell lysates. We infected BHK21 cells with MADV 1 for 24 h at an MOI = 1, then lysed the cells on ice for 30 min in a buffer containing 0.2% NP-40 or 2.5% SDS supplemented with BME. Untreated cell lysate samples contained 6.6 × 10^10^ PFU and an estimated 1 million cells. There were no plaques detected following either treatment (Table 1); however, as with fixatives, we observed cell loss in the detergent plus media-only controls at the first two dilutions in the series of plaque assays. We performed the same 13-day double passage experiment and observed no plaques or cell loss at any dilution (Table 2). Therefore, 0.2% NP40 and 2.5% SDS can inactivate MADV-infected cell lysates.

TRIzol and TRIzol LS are commercially-available acid-guanidinium-phenol reagents used to isolate RNA, DNA, and protein from cells and supernatants, respectively. To test whether TRIzol LS would inactivate EEEV-infected supernatants, we mixed stock virus and TRIzol LS at a ratio of 1 part virus to 4 parts TRIzol LS. This contained a total of 6.6 × 10^5^ PFU of MADV 1. For TRIzol, 1 mL of TRIzol was added directly to the wells of a 6-well plate containing BHK21 cells that had been infected for 24 h with MADV 1 at an MOI = 1. This corresponded to 6.6 × 10^10^ total PFU and 1 million cells. Both the TRIzol LS and TRIzol samples were incubated at room temperature for 15 min. We again observed cell loss due to TRIzol/TRIzol LS at the lowest plaque assay dilutions. There were no plaques from inactivated samples at dilutions where the cell monolayer was normal. The 13-day, two-passage experiments confirmed full inactivation of the samples by both TRIzol and TRIzol LS (Table 1 and Table 2). There was no cell loss, and no plaques formed.

UV inactivation can be an easy and rapid method to inactivate infectious samples; therefore, we assessed different amounts of UV radiation for the inactivation of MADV. 500 μL of stock virus was added to 6 well plates, resulting in a depth of 52 mm of supernatant containing 3.3 × 10^7^ PFU. Two rounds of 999 mJoules, one round of 999 mJoules, 250 mJoules, and 90 s of UV exposure all successfully inactivated MADV (Table 1). We did not observe cell loss in plaque assays of uninfected media exposed to two rounds of 999 mJoules and would have been able to detect any plaques that might have formed at the initial dilution. Therefore, we did not perform the 13-day double-passage experiment.

Heat inactivation is a common physical method of inactivation. We tested whether incubation of MADV at 95 °C effectively inactivated the virus. 200 μL of stock virus was incubated for 5, 10, or 15 min in a heat block set to 100 °C. This ensured a water temperature of 95 °C within a screw-cap tube, which was monitored with a thermometer to verify the correct temperature was achieved within each inactivation tube. MADV was fully inactivated after just 5 min of incubation (Table 1), indicating that heat is a rapid and efficient method for MADV inactivation.

Formalin cross-links primary amines and sulfhydryls within viral proteins, resulting in viral inactivation [14]. This could alter the structure of the viral protein, thereby compromising the ability of antibody reagents used in downstream procedures to detect the viral protein. UV inactivation, on the other hand, primarily inactivates viruses by inducing nucleotide dimerization within the viral genome, although free radicals generated by UV radiation can also react with amino acids and alter protein characteristics [15]. To validate that our inactivation procedures maintain key features of MADV and that the samples can be used effectively in different analytics procedures, we performed Western blots to probe for viral protein and RT-PCR to amplify genetic material for sequencing. MADV-infected supernatants were inactivated with 999 mJoules or 10% formalin, as described above, and removed from the BSL3. A fraction of each inactivated sample was lysed with a lysis buffer containing 0.5% NP-40, and the lysed and un-lysed samples, treated only with 2× Laemmli loading buffer, were separated on SDS-PAGE gels and transferred to PVDF membranes. We probed for E2 glycoprotein expression using a commercially-available rabbit polyclonal antibody against EEEV E2 protein. In both lysed and intact UV-inactivated virus samples, we detected several bands that we determined to be non-specific, as bands of the same molecular weight were also seen in an uninfected media-only control lane (Figure S1A). Furthermore, the anti-EEEV E2 antibody picked up a specific band corresponding to HA-tagged EEEV E2 that was expressed and immunoprecipitated from 293T cells, confirming the functionality of the antibody (Figure S1B). Therefore the anti-EEEV E2 antibody does not appear to be cross-reactive with MADV E2. We were unable to detect any bands in the formalin-treated samples. This is not surprising, given that during sample loading, we observed a separation of the virus-containing formalin fraction from the 2× Laemmli buffer within the well. There are likely incompatibilities between formalin and the running buffer or loading buffer that prevented proteins from moving electrophoretically through the SDS-PAGE gel. Additionally, we performed an alignment of the reference sequences for EEEV E2 (NP_740646.1) and MADV E2 (YP_009020589.1) and found the proteins to be only 87.9% identical at the amino acid level. This sequence divergence and associated changes in structure could prevent cross-reactivity of the antibody. We cannot rule out, however, that formalin treatment altered the E2 structure in a way that made the epitope unrecognizable by the rabbit antibody. Formalin-induced changes to domain III of the envelope (E) glycoprotein of the Japanese Encephalitis Virus that alter E antigenicity have been reported [16].

**Figure 3:**
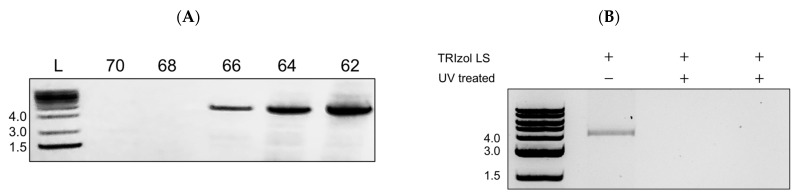
TRIzol-inactivated, but not UV-inactivated, MADV can be amplified by RT-PCR. (**A**) MADV stock was inactivated by TRIzol LS as described in methods and removed from the BSL3. Total RNA was isolated, followed by cDNA synthesis. The 5′ end of the MADV genome was amplified by PCR at the indicated primer annealing temperatures and separated on a 1% agarose gel. A band just above 4 kb, corresponding to the expected product size of 4022 bp and encompassing NSP1 and NSP2, was successfully detected at three annealing temperatures. (**B**) MADV-infected supernatants were inactivated with 999 mJoules as described in methods and removed from the BSL3. Total RNA was isolated using TRIzol LS, followed by cDNA synthesis and PCR amplification as described in B. Lanes 2 and 3 are technical replicates, and no product was amplified from UV-treated supernatants.

To validate that MADV genetic material can be detected and analyzed from samples inactivated by TRIzol LS, we performed RT-PCR followed by sequencing for viral genetic material. A sample of MADV stock virus was inactivated by TRIzol LS as described in the methods and removed from the BSL3. We extracted total RNA and synthesized complementary DNA (cDNA). A 4022 bp cDNA fragment, corresponding to the MADV non-structural proteins 1 and 2 (NSP1, NSP2), was successfully amplified by PCR (Figure 3A). We then purified the fragment from the gel and sent the sample for sequencing. When we ran the sequence results through a BLAST search, all of the hits mapped to sequences annotated as Madariaga or Eastern Equine Encephalitis Virus, with the strain not specified. These results validate that viral RNA from TRIzol LS-inactivated samples can be used in downstream applications and confirm the identity of our virus stock as Madariaga. To test whether UV inactivation damaged MADV genetic material, we attempted to amplify the same 4022 bp fragment from infected supernatants that had been inactivated with 999 mJoules. We isolated total RNA from UV-inactivated samples using TRIzol LS, as described above, followed by RT-PCR. The expected 4022 bp fragment was successfully amplified from TRIzol LS-treated, but not UV-treated, samples (Figure 3B). This indicates that the process of UV inactivation damages MADV genetic material such that it cannot be detected by PCR. UV radiation is expected to induce nucleotide dimerization within the MADV genome, which may prevent cDNA synthesis, and therefore there is no cDNA template of the MADV genome available for PCR amplification. It is important to note that while MADV genetic material can be removed from the BSL3, the EEEV genome is itself a Select Agent and cannot be removed from containment following TRIZol LS/TRIzol inactivation without additional fragmentation steps that render the genome non-infectious.

## 4. Discussion

Our results demonstrate that heat, formaldehyde-based fixatives, TRIzol/TRIzol LS, NP40 and SDS detergents, and UV radiation are effective inactivation methods for MADV (Table 1). Thirty minutes of incubation with formaldehyde-based fixatives successfully inactivated up to 6.6 × 10^10^ total PFU of MADV, which was the greatest amount of virus we tested. These fixatives were effective for both MADV-infected cell culture supernatants and infected cells. Fifteen minutes of incubation with TRIzol and TRIzol LS were sufficient to inactivate 6.6 × 10^10^ PFUs (1 million cells) and 6.6 × 10^5^ PFUs, respectively. Lysis buffers containing 0.2% NP40 or 2.5% SDS fully inactivated up to 6.6 × 10^10^ PFUs (1 million cells) within 30 min. Four different UV conditions (2 × 999 mJoules, 1 × 999 mJoules, 250 mJoules, 90 s) were able to inactivate 3.3 × 10^7^ PFUs, as well as just 5 min of incubation at 95 °C.

The process of inactivation could alter the characteristics of the virus, thereby complicating downstream analysis of inactivated samples. For example, the structure of viral proteins could be altered by formalin or UV inactivation, or viral genome integrity could be compromised by TRIZol LS treatment and subsequent RNA extraction. To confirm that our inactivation methods are compatible with downstream analyses, we performed Western blots and RT-PCR on inactivated samples. Our results confirm that TRIzol LS-inactivated MADV genetic material can be amplified by RT-PCR and sequenced (Figure 3A). On the other hand, UV inactivation is not compatible with downstream PCR analysis of MADV genetic material (Figure 3B). The antibody against EEEV E2 was not cross-reactive with MADV E2, and we were unable to confirm that UV or formalin-inactivated MADV can be analyzed for protein expression by Western blotting. Using a MADV-specific antibody may solve this issue. The inactivation methods described in this study will be useful for a wide range of applications and enable researchers to expand the types of experiments and analyses conducted with MADV and, by extension, EEEV.

Few inactivation protocols have been published for EEEV, but our results are consistent with previous findings on inactivation methods for other alphaviruses. Patterson et al. demonstrated that the Venezuelan Encephalitis Virus (VEEV) is fully inactivated by 10% formalin within 30 min at room temperature [17]. This group also tested 0.1% SDS but found this to be ineffective for VEEV inactivation. Here, we used a solution containing 2.5% SDS, which successfully inactivated MADV. This indicates that SDS-based inactivation is concentration-dependent and underscores the importance of testing multiple conditions. Our UV results fall within the range of doses reported to inactivate other alphaviruses. We demonstrate that MADV is inactivated by 26 mJoules/cm^2^, compared to 6 mJoules/cm^2^ for Sindbis virus [18] and 90 mJoules/cm^2^ for Chikungunya virus [19]. One study that tested EEEV specifically also found TRIzol LS to be effective [20], in agreement with our results. Overall, each of our inactivation methods is consistent with what has been demonstrated for related alphaviruses. To our knowledge, this is the first comprehensive study of inactivation methods for viruses in the Eastern Equine Encephalitis antigenic group.

It is important to consider factors that may affect the efficiency of these inactivation methods for MADV. The mechanism of UV inactivation of viruses occurs through UV-induced dimerization of nucleic acids. For MADV, this is the ssRNA genome. The optimal wavelength to induce RNA dimerization is 254 nm [21], which is the wavelength that our UV GeneLinker emits. Although proteins primarily absorb wavelengths of 280 nm, there is minor absorption at 254 nm, and proteins present in a sample can effectively reduce the UV delivered to nucleic acids. Previous studies have reported that higher amounts of protein, such as FBS or another cell culture serum, can decrease the efficacy of UV inactivation [22,23]. All of our infected supernatant samples contained 2% FBS. Although every UV condition we tested successfully inactivated MADV, researchers working with samples that contain higher serum content will need to confirm the suitability of the UV conditions reported here. Furthermore, increasing the depth of the infected supernatant, even if the total PFU remains the same, may reduce the efficacy of the lower mJoule conditions we tested. UV penetration decreases with increasing liquid depth, thus impacting incidence with virions further below the surface and diluting the effect of the radiation as the UV waves are absorbed by other molecules closer to the surface [21].

Researchers should note that intact EEEV genetic material is itself a Select Agent, as the positive-sense RNA genome can be translated into infectious virions and therefore cannot be removed from the BSL3 [24]. A cDNA clone of EEEV is not regulated and would overcome this restriction [24]. Our failed attempts to amplify a section of the UV-inactivated MADV samples by PCR suggest that UV fragments damage the genome such that the RNA itself would no longer be infectious. A study on UV-inactivation of SARS-CoV-2 similarly reported a UV-dose-dependent decrease in viral genome amplification by RT-PCR [25]. In contrast, we were able to amplify MADV by PCR using TRIzol-inactivated samples, indicating that while TRIzol inactivates the MADV virion, the RNA itself is likely still infectious and, when applied to EEEV, would need to undergo fragmentation prior to removal from the BSL3.

Here, we have validated a range of inactivation methods for EEEV using MADV in place of the select agent EEEV. To our knowledge, this is the first comprehensive study of heat, fixative, detergent, UV, and phenol-based inactivation methods for EEEV. Our findings will support researchers working with EEEV and related viruses by expanding the options for inactivation methods that are compatible with downstream applications. We anticipate that these protocols will advance the types of basic research that can be performed with EEEV, which will be necessary to address this emerging threat to public health.

## Figures and Tables

**Table 1:** Summary of inactivation results.

Treatment	Time/Dose	Supernatant/Cells	Virus: Input PFUs or Cells *	Inactivated: (Y/N)/Output PFU **
10% formalin	30 min	supernatant	6.6 × 10^5^	Y/0
	60 min	supernatant	6.6 × 10^5^	Y/0
4% PFA	30 min	supernatant	6.6 × 10^5^	Y/0
	60 min	supernatant	6.6 ×10^5^	Y/0
TRIzol LS	15 min	supernatant	6.6 × 10^5^	Y/0
UV	90 s	supernatant	3.3 × 10^7^	Y/0
	250 mJoule	supernatant	3.3 × 10^7^	Y/0
	1 × 999 mJoule	supernatant	3.3 × 10^7^	Y/0
	2 × 999 mJoule	supernatant	3.3 × 10^7^	Y/0
Heat	5 min	supernatant	1.3 × 10^7^	Y/0
	10 min	supernatant	1.3 × 10^7^	Y/0
	15 min	supernatant	1.3 × 10^7^	Y/0
10% formalin	60 min	cell pellet	1 million	Y/0
4% PFA	60 min	cell pellet	1 million	Y/0
TRIzol	15 min	cells	1 million	Y/0
2.5% SDS	30 min	cells	1 million	Y/0
0.2% NP40	30 min	cells	1 million	Y/0
None	N/A	cells	24 h infection, MOI = 1	8 × 10^6^–6.6 × 10^10^
None	N/A	supernatant	24 h infection, MOI = 1	1 × 10^8^–4 × 10^9^

* Input PFUs/cells: the total number of PFUs that were inactivated in each condition. In cases of cell pellet or cell inactivation, the input number of PFUs and the corresponding number of cells are both listed. ** Inactivation status indicated as Y (yes) or N (no) and the output PFUs detected following inactivation.

**Table 2:** Summary of results of double-passage 13-day experiments.

Treatment	Time	Supernatant/Cells	Day 13 PFU/mL	Inactivated Y/N
10% formalin	60 min	cell pellet	0	Y
4% PFA	60 min	cell pellet	0	Y
TRIzol	15 min	cells	0	Y
TRIzol LS	15 min	supernatant	0	Y
2.5% SDS	30 min	cell pellet	0	Y
0.2% NP40	30 min	cell pellet	0	Y
10% formalin	60 min	supernatant	0	Y
4% PFA	60 min	supernatant	0	Y

## Data Availability

The data in this study are available upon written request from the corresponding author (maudry.laurent-rolle@yale.edu).

## Supplementary Materials

The following supporting information can be downloaded at: https://www.mdpi.com/article/10.3390/v16020206/s1, Figure S1: Anti-EEEV E2 antibody is not cross-reactive with MADV E2 protein.
