# Supplementary material for: Comprehensive Assessment of Inactivation Methods for Madariaga Virus
Source: Viruses. 2024 Jan 30;16(2):206. doi: 10.3390/v16020206 (PMC10892135; doi:10.3390/v16020206)
Supplement: Supplementary file 1 [file viruses-16-00206-s001.zip › viruses-2708010-supplementary.pdf]

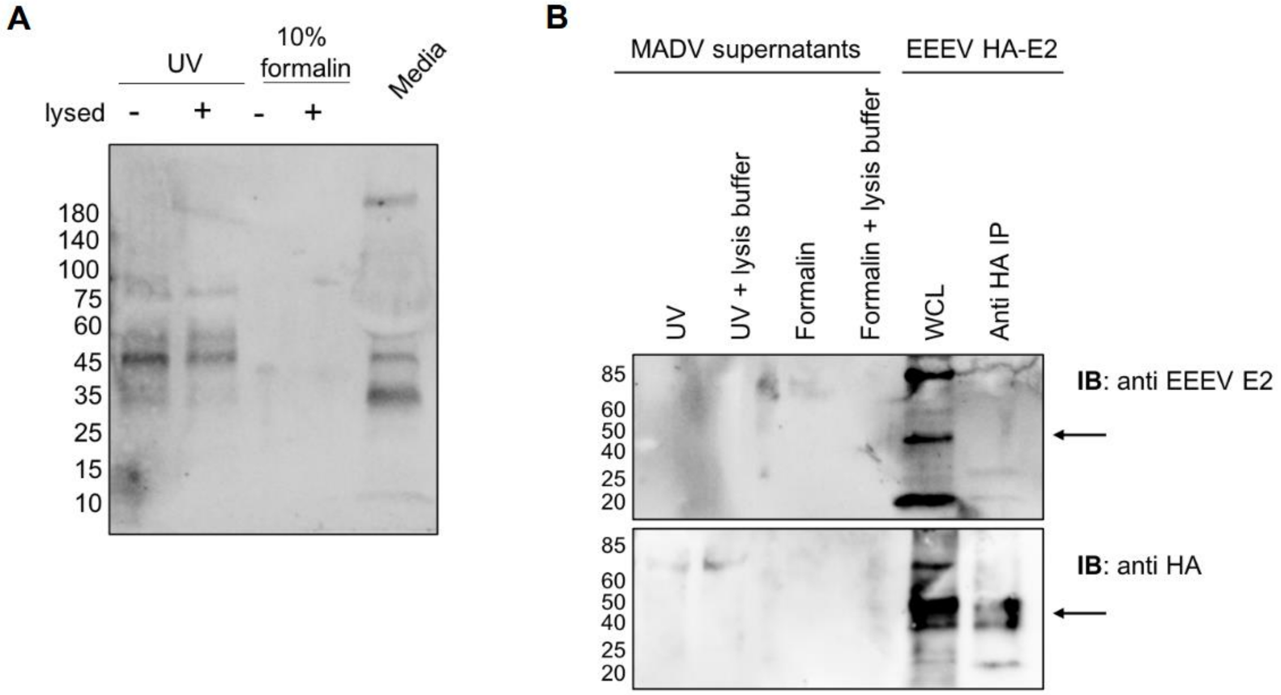

Figure S1. Anti-EEEV E2 antibody is not cross-reactive with MADV E2 protein. (**A**) The bands detected in the UV-treated samples are of the same molecular weight as bands in the uninfected media lane, indicating non-specific antibody binding. No cross-reactive proteins were detected in the formalin-inactivated samples. MADV stock was UV-inactivated with 999 mJoules or inactivated with 10% formalin and removed from the BSL3. Samples were then either lysed with a lysis buffer containing 0.5% NP-40, or left untreated, and separated on an SDS-PAGE gel. Proteins were transferred to a PVDF membrane and blotted for MADV envelope E2 protein using a commercially-available antibody against the EEEV E2. (**B**) Same samples as (**A**) but re-run with whole cell lysate and IP of EEEV E2 HA-tagged protein. Probing with the anti-EEEV E2 antibody detected a band at the expected molecular weight of about 40-50 kDa (arrows) in the whole cell extract from transfected cells. Probing for HA confirmed expression of EEV E2 HA-tagged protein. It is unclear why we did not observe a band in the IP lane with the anti-EEEV E2 antibody. From the anti-HA blot there is grossly less E2 in the IP lane using the HA antibody. There may be a difference in antibody sensitivity between the anti-HA (can be used at dilution up to 1:5000) and anti-EEEV E2 antibody (1:1000 maximum dilution).
